# A rapid facility-level assessment of oxygen systems in 39 low-income and middle-income countries: a cross-sectional study

**DOI:** 10.1016/S2214-109X(24)00561-8

**Published:** 2025-02-27

**Authors:** Nadir Ijaz, Tamsin Lee, Nicholas Furtado, Emilie Macher, Zipporah Muitheri, Benjamin J Park, David Lowrance

**Affiliations:** Yale University School of Medicine, New Haven, CT, USA; The Global Fund to Fight AIDS, Tuberculosis, and Malaria, Geneva, Switzerland; The Global Fund to Fight AIDS, Tuberculosis, and Malaria, Geneva, Switzerland; The Global Fund to Fight AIDS, Tuberculosis, and Malaria, Geneva, Switzerland; The Global Fund to Fight AIDS, Tuberculosis, and Malaria, Geneva, Switzerland; The Global Fund to Fight AIDS, Tuberculosis, and Malaria, Geneva, Switzerland; The Global Fund to Fight AIDS, Tuberculosis, and Malaria, Geneva, Switzerland

## Abstract

**Background:**

Unequal access to medical oxygen is a key driver of global inequities in morbidity and mortality. We aimed to describe reliable oxygen availability (ie, whether availability is uninterrupted) and functional availability (ie, whether oxygen system components are in working order) in 39 low-income and middle-income countries and to compare across WHO subregions.

**Methods:**

We report cross-sectional survey data from primary, secondary, and tertiary level health facilities across six WHO subregions. Facilities were selected via purposive and stratified random sampling. Data collectors visited facilities from September 2022, to February 2023, to administer a standardised questionnaire to facility leadership. All approached facilities responded. Questions assessed reliable oxygen availability over the preceding 3 months and the functional availability of system components: oxygen sources (ie, cylinders, concentrators, plants, and liquid oxygen), distribution systems (ie, piping, cylinder transport, and respiratory tubing), delivery devices (ie, nasal interfaces, face masks, and advanced modalities), monitoring devices (ie, pulse oximeters and multiparameter monitors), and quality assurance (ie, oxygen concentration control and maintenance schedule). We report descriptive statistics and compare across subregions using χ^2^ and Fisher exact tests.

**Findings:**

Of 2884 surveyed facilities, 304 (24·5%) of 1241 primary facilities, 558 (52·4%) of 1064 secondary facilities, and 387 (66·8%) of 579 tertiary facilities reported reliable oxygen availability. Facilities across levels and subregions lacked system components, with statistically significant (p<0·05) differences in functional availability of all oxygen system components across subregions at all levels. For example, functional availability of cylinders ranged from 56·7% to 100·0%, piping from 7·5% to 94·6%, nasal cannulae from 56·3% to 96·4%, and pulse oximeters ranged from 47·8% to 96·4%, depending on level and subregion.

**Interpretation:**

Reliable oxygen availability was low across facility levels and subregions. There were significant disparities in the functional availability of oxygen system components across subregions, with important implications for global health equity and financing.

**Funding:**

The Global Fund to Fight AIDS, Tuberculosis, and Malaria; the Yale National Clinician Scholars Program; the US National Center for Advancing Translational Sciences; the US National Heart, Lung, and Blood Institute; and the Yale Institute for Global Health.

## Introduction

Gaps in oxygen access have been documented in low-income and middle-income countries (LMICs) for decades and contribute to the deaths of an estimated 9 million people in LMICs each year from conditions that could require medical oxygen.^1-3^ Assessments from multiple LMIC contexts have previously reported high levels of oxygen unavailability,^4^ inadequate clinical staff training in oxygen therapy provision,^5^ and high proportions of patients who are hypoxaemic not receiving timely oxygen therapy.^6,7^ The COVID-19 pandemic further highlighted these pre-existing gaps in oxygen access as drivers of global inequalities in morbidity and mortality.^2,8^ Country-led quality assessments of COVID-19 treatment centres in LMICs revealed major oxygen gaps, high levels of equipment scarcity and non-functionality, and regional disparities.^9-16^

Oxygen systems require the coordination of multiple system components—including oxygen sources, distribution, delivery, monitoring devices, and quality assurance systems—that remain poorly characterised across LMICs.^17,18^ Most oxygen system assessments have focused on single countries, subnational administrative units, or facility types (eg, COVID-19 treatment centres).^9-16^ Few surveys assess whether oxygen availability is uninterrupted over a period of time (what we refer to as reliable availability).^19^ In addition, surveys often characterise oxygen systems solely by taking inventory of a subset of oxygen sources (eg, concentrators or cylinders), which could leave other crucial system components unmeasured. Facility assessment tools rarely parse the availability of specific system components or assess whether they are actually functional (functional availability).^19,20^ To the best of our knowledge, there has been no rigorous, standardised, multinational, facility-level assessment to measure reliable oxygen availability and the functional availability of oxygen system components across LMICs to date.

In this Article, we expand global knowledge about the status of oxygen systems in LMICs using data from a multinational, cross-sectional, facility-level survey of oxygen systems conducted by The Global Fund to Fight AIDS, Tuberculosis, and Malaria. Specifically, we aimed to describe facility-level reliable oxygen availability and the functional availability of specific types of oxygen sources, distribution systems, delivery devices, and hypoxaemia monitoring devices, and to compare these across WHO subregions.

## Methods

### Study design and data source

This multinational, cross-sectional survey was conducted in 39 LMICs as part of the Supply Chain and Health Services Spot Check (Spot Check) questionnaire administered by The Global Fund. The primary goal of the Spot Check questionnaire was to internally evaluate the continuity of essential health services and commodities during the COVID-19 pandemic. The Spot Check questionnaire was done in three rounds, with iterative refinements in survey questions and sampling methods after the first two rounds. This Article focuses on data collected during the third round of Spot Check data collection from September 2022, to February 2023 (see appendix 2 p 6 for specific range of time during which data collection occurred by geographical region). This study was deemed not human participants research by the Yale Institutional Review Board (2000038938).

### Participants

**Figure 1:**
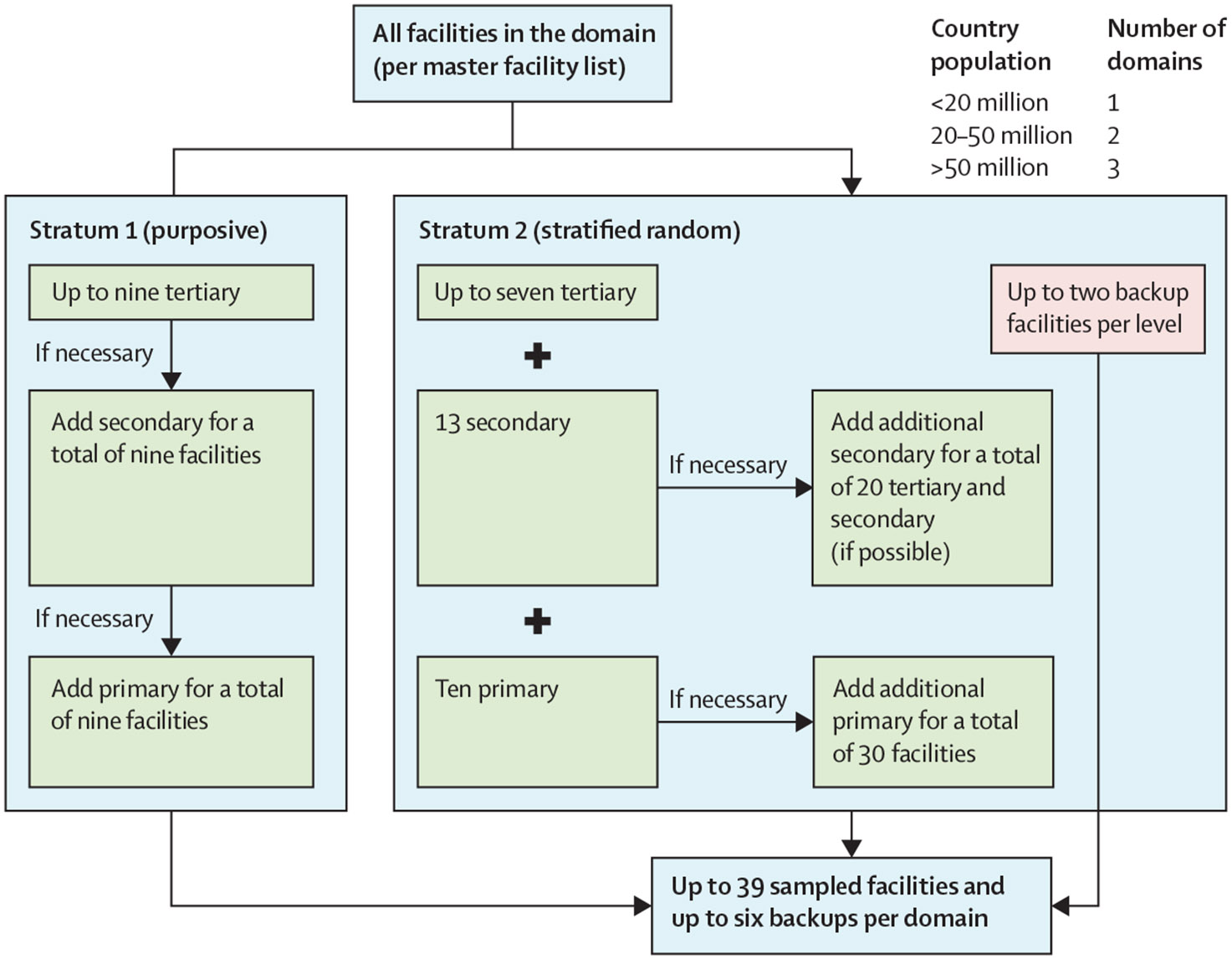
Sampling process Up to 39 facilities (with up to six backup facilities) were selected per geographical domain by purposive and stratified random sampling across primary-care, secondary-care, and tertiary-care levels.

Countries were selected based on various financial and operational factors. Each country was divided into one, two, or three geographical domains based on their populations (figure 1). Health facilities were sampled from each country’s master facility list provided by the ministry of health or equivalent. Each master facility list provided information on all recognised public sector health facilities in its country, including facility name, care level, and geographical location. Sampling within each geographical domain had two primary strata: a stratum of nine panel facilities known to receive support from The Global Fund purposively selected as a panel and a stratum of all other eligible non-panel facilities in the country, from which 30 facilities were randomly selected (via a computer program). Using this approach, at least 39 facilities were sampled per geographical domain (figure 1). Two backup facilities of each level were also randomly selected to replace facilities that could not be assessed for security reasons and were therefore excluded. In domains with more excluded facilities than backup facilities available to replace them, fewer than 39 facilities were assessed (table 1).

Panel facilities were purposively selected based on their management of patients with COVID-19 since the start of the pandemic and their receipt, or expected receipt, of support from The Global Fund through the COVID-19 Response Mechanism. Selection of panel facilities began with tertiary facilities. If there were fewer than nine tertiary facilities, additional facilities from the secondary-care level were sampled, and, if necessary, facilities from the primary-care level were sampled (figure 1).

Selection of non-panel facilities was done using stratified random sampling, with the secondary level of stratification based on facility level: primary (eg, health centres and small clinics), secondary (eg, district general hospitals), and tertiary (eg, specialty referral hospitals). Within stratum two, we sampled 30 facilities per geographical domain. The proportions of primary, secondary, and tertiary facilities sampled within each geographical domain depended on the total number of secondary and tertiary facilities in the non-panel stratum (figure 1).

### Procedures

The Global Fund used three external data service providers (IQVIA, Palladium, and Klynveld Pear Marwick Goerdeler) to facilitate data collection. Data service providers trained one data collection team comprised of public health monitoring and evaluation specialists per surveyed country, led by a team leader. Although teams had variable previous technical experience with oxygen systems, all team leaders and many data collectors had experience with Spot Check surveys from earlier survey rounds and were therefore familiar with general Spot Check methods for at least 6 months. Data service providers also conducted online training for data collection teams and followed up with intensive in-person training for teams showing inadequate competency on a knowledge and skills assessment. Data collectors travelled to each survey site to administer the questionnaire among facility staff leadership. Although the primary respondent was the Officer in Charge of the health facility, if the Officer indicated another individual would have more information about specific items, these individuals were approached. All responses were recorded in an electronic data entry form in real time.

We used variables of interest from the facility information and oxygen readiness sections of the Spot Check questionnaire. Facility information included geographical location; the presence of services for HIV, tuberculosis, malaria, and COVID-19; and facility-care level (ie, primary, secondary, or tertiary). Oxygen readiness variables included facility-level availability of oxygen therapy (does the facility offer oxygen therapy and related services?) and periods of oxygen unavailability over the preceding three months (has oxygen been unavailable at this facility at any time over the past three months for any reason?). For all facilities offering oxygen therapy, the survey also assessed the availability and functionality of the following oxygen system components, as reported by the respondent on the day of the survey: source (ie, cylinders, concentrators, pressure-swing-adsorption plants, and liquid oxygen), distribution (ie, central or subcentral piping, cylinder transport, and respiratory tubing), delivery devices (ie, nasal cannula, nasal catheter, face mask, and invasive mechanical ventilation), and monitoring devices (ie, pulse oximeters and multiparameter monitors). Facilities were also asked about the availability (but not the functionality) of non-invasive mechanical ventilation devices and whether they implemented a maintenance schedule for critical oxygen-generating equipment and oxygen concentration control. The full questionnaire is available in appendix 2 (pp 7-9).

We defined reliable oxygen availability as the facility-level availability of oxygen therapy without any periods of oxygen unavailability reported over the preceding three months, functional availability of an oxygen system component as the facility report of its availability and its functionality at the time of the survey, and bulk oxygen source as either a pressure-swing-adsorption plant or liquid oxygen.

### Statistical analysis

First, we reported frequencies and proportions for binary or categorical variables of interest. We stratified our sample by facility level (ie, primary, secondary, or tertiary) and WHO subregion (ie, Africa D, Africa E, Eastern Mediterranean D, South-East Asian B, South-East Asian D, and Western Pacific B; table 1). These subregions have been previously defined on the basis of child and adult mortality, with the letter B representing the lowest mortality strata and E the highest mortality strata.^21^ Second, we used the χ^2^ and Fisher exact tests of independence to respectively identify the statistically significant differences (with a two-sided α=0·05) in reliable oxygen availability and the functional availability of oxygen system components across WHO subregions for primary-care, secondary-care, and tertiary-care level facilities.

Because of the purposively sampled panel facilities, the overall sampling strategy was not fully random, and our sample was not representative at the country or regional or provincial level. In addition, because of oversampling at the secondary-care and tertiary-care levels, with fewer facilities at those levels, the sampling ratio for secondary-care and tertiary-care levels was much higher (100% for some countries) than for the primary-care level (as low as 0·18%). For these reasons, we report only unweighted estimates in our analysis and do not report on the country level. We used Stata 17.0 SE for data analysis.

### Role of the funding source

The Global Fund had a role in study design, data collection, data analysis, data interpretation, and writing of the report. The other funders had no role in study design, data collection, data analysis, data interpretation, or writing of the report.

## Results

**Figure 2:**
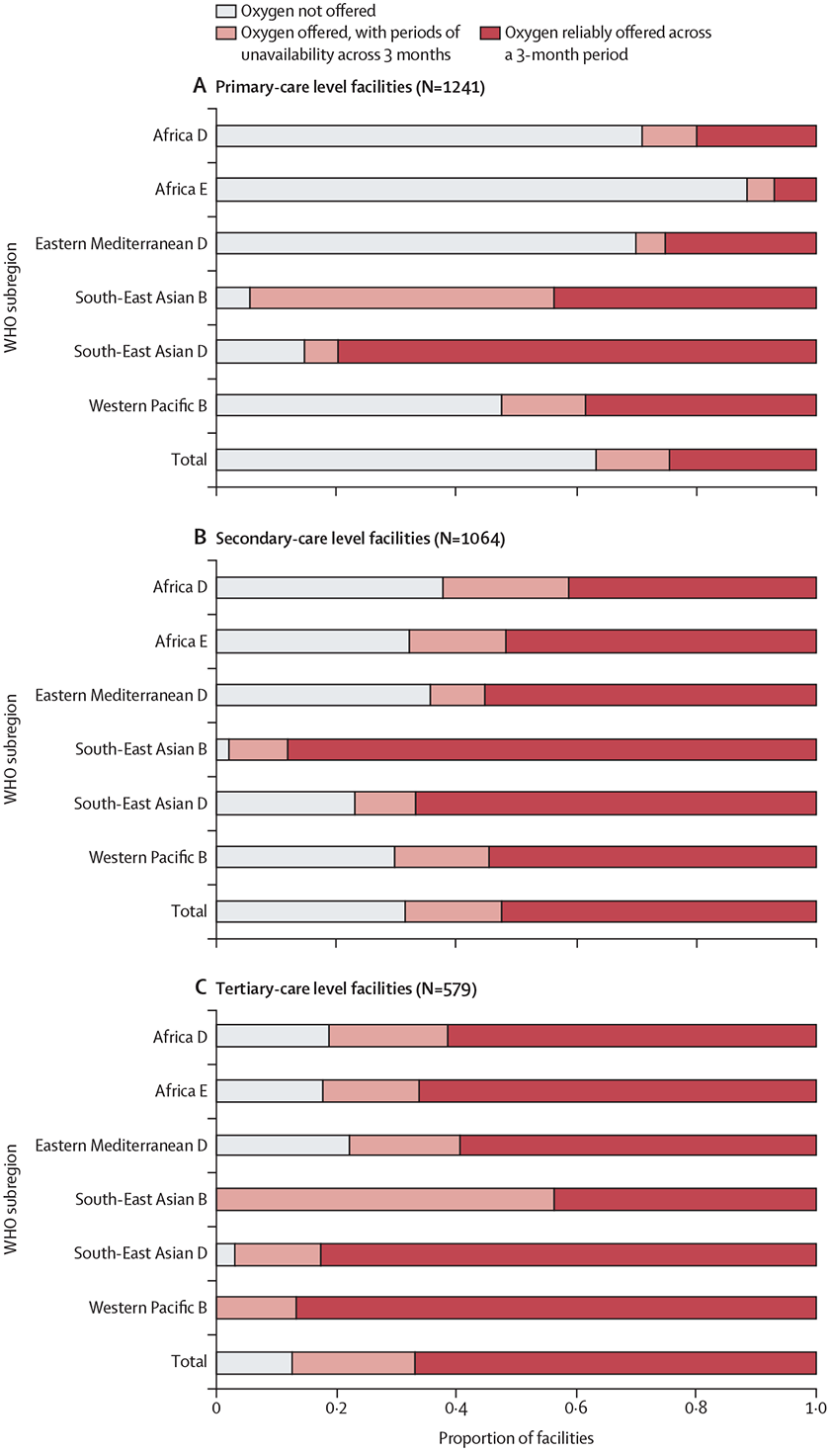
Reliable oxygen availability by facility-care level and WHO subregion

Our sample included 2884 health facilities in 39 countries, including 1241 primary-care, 1064 secondary-care, and 579 tertiary-care level facilities. Most sampled facilities were in the African subregions (table 1). Of sampled facilities, 1689 (58·6%) of 2884 reported offering oxygen therapy, including 454 (36·6%) of 1241 primary-care, 729 (68·5%) of 1064 secondary-care, and 506 (87·4%) of 579 tertiary-care level facilities. Facilities in the Africa E subregion were the least likely to offer oxygen therapy (p<0·0001 for all pairwise comparisons with other subregions). Of facilities offering oxygen therapy, 150 (33·0%) of 454 primary-care, 171 (23·5%) of 729 secondary-care, and 119 (23·5%) of 506 tertiary-care level facilities reported having at least one period of oxygen unavailability over the preceding three months. Only 304 (24·5%) of 1241 primary-care, 558 (52·4%) of 1064 secondary-care, and 387 (66·8%) of 579 tertiary-care level facilities reported reliable oxygen availability over the preceding three months (figure 2).

Among the 454 (36·6%) of 1241 primary-care level facilities offering oxygen, 330 (72·7%) had functional cylinders and 303 (66·7%) had functional concentrators (table 2). A bulk oxygen source was present in 195 (43·0%) primary-care level facilities. Facilities at primary-care level in Western Pacific B were the least likely to have functional cylinders (p<0·05 for all pairwise comparisons except with Africa D and Africa E), concentrators (p<0·05 for all pairwise comparisons with other subregions), or pressure-swing-adsorption plants (p<0·05 for all pairwise comparisons except with Eastern Mediterranean D). 152 (33·5%) primary-care level facilities had central or subcentral piping, and more than 40% did not have a cylinder transport system and respiratory tubing. Face masks were the most commonly available delivery device, present in 370 (81·5%) primary-care level facilities overall and almost universally present in South-East Asian B facilities. Pulse oximetry was available in 340 (74·9%) facilities and least likely to be available in Western Pacific B facilities (32 [47·8%] of 67). Both oxygen concentration control and equipment maintenance schedules were present in approximately 70% of primary-care level facilities. There were statistically significant differences in the functional availability of each oxygen system component across subregions (table 2).

Among the 729 (68·5%) of 1064 secondary-care level facilities offering oxygen, 608 (83·4%) had functional cylinders and 598 (82·0%) had functional concentrators (table 3). Almost all South-East Asian D facilities at secondary-care level had functional cylinders. A bulk oxygen source was present in 341 (46·8%) secondary-care level facilities. While almost two-fifths of secondary-care level facilities had central or subcentral piping, this proportion was much higher for South-East Asian B facilities (40 [80·0%] of 50) than for facilities in other subregions (p<0·05 for all pairwise comparisons except with South-East Asian D). More than one-third of secondary-care level facilities did not have respiratory tubing, although this was almost universally available in South-East Asian B. Face masks were the most commonly available oxygen delivery device in every subregion. Pulse oximetry was available in 606 (83·1%) secondary-care level facilities and least likely to be available in Eastern Mediterranean D facilities. More than 75% of all secondary-care level facilities had oxygen concentration control and equipment maintenance schedules, including almost all South-East Asian B facilities (table 3).

506 (87·4%) of 579 tertiary-care level facilities offered oxygen, of which 458 (90·5%) had functional cylinders and 448 (88·5%) had functional concentrators (table 4). Almost three-quarters of tertiary-care level facilities had a bulk oxygen source. Almost all South-East Asian B facilities at tertiary-care level had liquid oxygen availability (53 [96·4%] of 55 facilities), whereas South-East Asian D facilities were most likely to have pressure-swing-adsorption plants (55 [82·1%] of 67 facilities; p<0·05 for all pairwise comparisons with other subregions). Overall, only 325 (64·2%) tertiary-care level facilities had central or subcentral piping; this included almost all South-East Asian B facilities but only 16 (34·8%) of 46 Eastern Mediterranean D facilities. Cylinder transport systems and respiratory tubing were also almost universally available in South-East Asian B facilities but lacking in almost half of Eastern Mediterranean D facilities. Face masks were available in 465 (91·9%) of all tertiary-care level facilities. Invasive mechanical ventilation was available in 48 (87·3%) of 55 South-East Asian B facilities but only 18 (39·1%) of 46 Eastern Mediterranean D facilities. Pulse oximetry was available in more than 90% of tertiary-care level facilities and least likely to be available in Western Pacific B facilities. Multiparameter monitors were available in 50 (90·9%) of 55 South-East Asian B facilities but only in 24 (52·2%) of 46 Eastern Mediterranean D facilities. Overall, 436 (86·2%) and 437 (86·4%) of 506 tertiary-care level facilities had systems for oxygen concentration control and an equipment maintenance schedule, respectively (table 4).

## Discussion

In this cross-sectional survey of 2884 facilities across 39 LMICs, only 58·6% reported offering oxygen therapy and, among these facilities, less than half had reliable oxygen availability over the preceding 3 months. Even among facilities offering oxygen therapy, oxygen system components were often missing across care levels and WHO subregions. Reliable oxygen availability and the functional availability of specific system components were different and unequal across WHO subregions for almost all system components, highlighting important global health inequalities and community vulnerability.

Given oxygen’s status as an essential medicine,^22^ these findings are especially humbling. Both as a part of strengthening universal health coverage and pandemic preparedness and response, WHO guidelines recognise the need for basic oxygen-related equipment, such as oxygen cylinders, respiratory tubing, face masks, pulse oximeters, and multiparameter monitors at all care levels.^23-25^ Despite this, we found substantial gaps in the functional availability of even these basic oxygen system components across subregions and facility-care levels. Our findings are supported by previous, smaller-scale, country-level assessments identifying similar gaps in the availability of functional oxygen system components.^9-16^

To the best of our knowledge, this is the first study to not only assess oxygen availability but also to compare the availability of specific, functional system components across many countries in six WHO subregions using standardised methods. We find that the functional availability of all system components varied significantly across WHO subregions at each facility-care level. Possible underlying mechanisms for these inequalities could be structural (eg, disparities in access to power grids and road networks),^26,27^ financial (eg, public health funding for vertical disease-specific programmes *vs* horizontal programmes strengthening health systems),^28^ technical (eg, historically little explicit emphasis on medical oxygen as part of pandemic preparedness and response implementation),^29^ and political (eg, little political prioritisation of oxygen systems development).^30^ Future research should examine which combinations of these and other historical factors have driven global inequalities in oxygen systems and capacity.

Comparing the functional availability of system components across subregions reveals important differences in oxygen system phenotype that might be overlooked in facility assessments focused solely on oxygen access. For example, all the tertiary-care level facilities in the two B mortality stratum subregions reported offering oxygen therapy. However, tertiary-care level oxygen systems in these two relatively high-performing subregions look quite different. In South-East Asian B, 96·4% of tertiary care level facilities reported having a functional bulk oxygen source, whereas in Western Pacific B, only half of tertiary-care level facilities reported the same, implying much more reliance on small-volume oxygen sources, such as cylinders and concentrators. Similarly, the proportion of secondary-care level facilities in the South-East Asian D subregion with central or subcentral piping was almost twice as high as in the Africa D and Eastern Mediterranean D subregions. These differences could represent multiple pathways to functional oxygen systems, or they could be the drivers of differences in reliable oxygen availability between these subregions.

Among oxygen system components, pulse oximetry availability was relatively high across facility levels and subregions. This finding is encouraging and might be due to the considerable research and efforts in pulse oximetry implementation in LMICs since 2014. However, although pulse oximetry is the global standard for detecting and monitoring hypoxaemia,^24^ 25·0% of primary-care, 16·9% of secondary-care, and 9·7% of tertiary-care level facilities lacked functional pulse oximeters. Furthermore, we did not assess patient-level availability (eg, availability in different service delivery areas within a facility and for different types of patients), which is likely substantially lower than facility-level availability.

Our findings should be interpreted in the context of the following limitations. First, our survey is based on facility report rather than direct verification of oxygen system component availability and functionality. Although respondents could have reported higher levels of functional availability than actually existed, the use of in-person site visits for data collection might have mitigated this bias. In addition, we did not collect detailed data on oxygen system components for sampled facilities that did not offer oxygen therapy, some of which could have had system components that were non-functional, resulting in a lack of oxygen. Future research should examine the functional status of any oxygen system components in these types of facilities.

Second, in our analysis, we assigned facility-care levels as identified by facility leadership. These care levels were often defined differently across countries and subregions, and the primary-care level often included both rural health posts as well as health centres, which provide different sublevels (ie, different types of facilities within the same level) of care. Our finding that 43·0% of primary-care facilities had a bulk oxygen source suggests that some of these facilities might have been operating as higher-level referral centres or could have misinterpreted survey questions. We were unable to parse the differences among these different care levels and sublevels. Future research should examine these differences and work toward standardisation of care level definitions as they relate to oxygen systems.

Third, since the Spot Check questionnaire was administered in different contexts with potentially different understandings of oxygen system components, some survey questions might have been interpreted differently by respondents. Although most items were unambiguous, the survey did not specify whether cylinder transport was inter-facility or intra-facility. The nuances of different possible interpretations of such system components should be considered, and future surveys should ideally be validated to ensure questions are interpreted the same way across various contexts.

Fourth, some countries included private health facilities or those affiliated with non-governmental organisations on their master facility lists. Although these facility types might comprise some of our sample, we expect this to be a small proportion of our total sampled facilities. We focused on differences in oxygen systems by geographical subregion; future studies might find equally compelling differences between sectors of the health-care system within a particular country.

Fifth, our analysis was limited to the subregional level, and we were unable to report country-level statistics. There are also components integral to oxygen access that were not included in our survey (eg, power supply). Our study serves as a starting point for examining global disparities, and similar methods could be used to evaluate for disparities at the country or sub-country level and for other components of oxygen systems.

In summary, we report a staggeringly low frequency of reliable oxygen availability among LMIC facilities, with even facilities offering oxygen often not having basic system components. Differences across WHO subregions highlight long-standing global inequalities that not only hold importance for pandemic preparedness but also for the equitable and meaningful care of all individuals with oxygen-requiring conditions globally. These findings suggest how global health financing of oxygen systems, whether linked to universal health coverage, pandemic preparedness and response, or vertical disease policy and programme objectives, could have profound, potentially lasting impacts on global morbidity and mortality. Future research should focus on refining our methods of oxygen system measurement, elucidating the structural, economic, technical, and political forces contributing to global inequalities, and identifying strategies for the sustainable implementation of life-saving oxygen systems and services in LMICs.

## Supplementary Material

Supplementary appendix 1

Supplementary Appendix 3

Supplementary Appendix 2

## Figures and Tables

**Table 1:** Sampled facilities by WHO subregion, country, and facility-care level

	Primary	Secondary	Tertiary
Africa D	473	311	156
Angola	52	22	4
Benin	16	18	5
Burkina Faso	27	44	6
Cameroon	58	9	11
Chad	22	9	6
Ghana	51	22	5
Guinea	10	26	3
Liberia	13	11	15
Madagascar	22	32	24
Mali	61	7	5
Niger	49	16	8
Nigeria	39	54	42
Senegal	7	22	9
Sierra Leone	19	13	7
Togo	27	6	6
Africa E	336	391	164
Burundi	23	11	5
Côté d’Ivoire	22	49	7
Democratic Republic of the Congo	32	63	22
Ethiopia	42	50	25
Kenya	26	60	31
Lesotho	24	15	0
Malawi	17	18	4
Mozambique	29	33	16
Tanzania	77	26	8
Uganda	19	42	17
Zambia	15	10	14
Zimbabwe	10	14	15
Eastern Mediterranean D	107	107	59
Pakistan	43	49	25
Somalia	24	12	3
South Sudan	17	12	10
Sudan	23	34	21
South-East Asian B	128	51	55
Indonesia	78	6	33
Thailand	50	45	22
South-East Asian D	69	96	69
Bangladesh	38	55	24
India	31	41	45
Western Pacific B	128	108	76
Cambodia	15	11	13
Papua New Guinea	24	13	2
Philippines	44	39	34
Viet Nam	45	45	27

**Table 2:** Availability of functional oxygen system components in 454 primary-care level facilities that offer oxygen therapy, by WHO subregion

	Africa D (n=137)	Africa E (n=38)	Eastern Mediterranean D (n=32)	South-East Asian B (n=121)	South-East Asian D (n=59)	Western Pacific B (n=67)	Total (n=454)	p value
Oxygen sources								
Cylinders	81 (59·1%)	25 (65·8%)	27 (84·4%)	103 (85·1%)	56 (94·9%)	38 (56·7%)	330 (72·7%)	<0·0001
Concentrators	100 (73·0%)	28 (73·7%)	18 (56·3%)	99 (81·8%)	40 (67·8%)	18 (26·9%)	303 (66·7%)	<0·0001
Any bulk oxygen source*	66 (48·2%)	14 (36·8%)	12 (37·5%)	69 (57·0%)	19 (32·2%)	15 (22·4%)	195 (43·0%)	<0·0001
Pressure-swing-adsorption plants	45 (32·8%)	11 (28·9%)	4 (12·5%)	66 (54·5%)	13 (22·0%)	5 (7·5%)	144 (31·7%)	<0·0001
Liquid oxygen	53 (38·7%)	7 (18·4%)	11 (34·4%)	65 (53·7%)	11 (18·6%)	14 (20·9%)	161 (35·5%)	<0·0001
Oxygen distribution systems								
Central or subcentral piping	52 (38·0%)	8 (21·1%)	5 (15·6%)	65 (53·7%)	17 (28·8%)	5 (7·5%)	152 (33·5%)	<0·0001
Cylinder transport	88 (64·2%)	19 (50·0%)	19 (59·4%)	80 (66·1%)	28 (47·5%)	18 (26·9%)	252 (55·5%)	<0·0001
Respiratory tubing	80 (58·4%)	16 (42·1%)	13 (40·6%)	108 (89·3%)	23 (39·0%)	24 (35·8%)	264 (58·1%)	<0·0001
Oxygen delivery devices								
Nasal cannula	98 (71·5%)	29 (76·3%)	18 (56·3%)	107 (88·4%)	36 (61·0%)	39 (58·2%)	327 (72·0%)	<0·0001
Nasal catheter	69 (50·4%)	24 (63·2%)	15 (46·9%)	68 (56·2%)	19 (32·2%)	37 (55·2%)	232 (51·1%)	0·026
Face mask	99 (72·3%)	30 (78·9%)	28 (87·5%)	118 (97·5%)	48 (81·4%)	47 (70·1%)	370 (81·5%)	<0·0001
Continuous positive airway pressure†	27 (19·7%)	4 (10·5%)	6 (18·8%)	23 (19·0%)	5 (8·5%)	4 (6·0%)	69 (15·2%)	0·042
Bilevel positive airway pressure†	38 (27·7%)	8 (21·1%)	5 (15·6%)	50 (41·3%)	7 (11·9%)	5 (7·5%)	113 (24·9%)	<0·0001
Invasive mechanical ventilation	65 (47·4%)	14 (36·8%)	9 (28·1%)	55 (45·5%)	7 (11·9%)	4 (6·0%)	154 (33·9%)	<0·0001
Monitoring devices								
Pulse oximeter	94 (68·6%)	30 (78·9%)	22 (68·8%)	116 (95·9%)	46 (78·0%)	32 (47·8%)	340 (74·9%)	<0·0001
Multiparameter monitor	77 (56·2%)	16 (42·1%)	9 (28·1%)	74 (61·2%)	8 (13·6%)	6 (9·0%)	190 (41·9%)	<0·0001
Quality assurance systems								
Concentration control	85 (62·0%)	24 (63·2%)	22 (68·8%)	102 (84·3%)	38 (64·4%)	47 (70·1%)	318 (70·0%)	0·0019
Maintenance schedule	100 (73·0%)	20 (52·6%)	18 (56·3%)	104 (86·0%)	37 (62·7%)	43 (64·2%)	322 (70·9%)	0·0004

Data are n (%). *Bulk oxygen source refers to either pressure-swing-adsorption plants or liquid oxygen. †For continuous positive airway pressure and bilevel positive airway pressure, only availability (and not equipment functionality) was assessed.

**Table 3:** Availability of functional oxygen system components in 729 secondary-care level facilities that offer oxygen therapy, by WHO subregion

	Africa D (n=194)	Africa E (n=266)	Eastern Mediterranean D (n=69)	South-East Asian B (n=50)	South-East Asian D (n=74)	Western Pacific B (n=76)	Total (n=729)	p value
Oxygen sources								
Cylinders	141 (72·7%)	235 (88·3%)	58 (84·1%)	44 (88·0%)	73 (98·6%)	57 (75·0%)	608 (83·4%)	<0·0001
Concentrators	165 (85·1%)	240 (90·2%)	47 (68·1%)	45 (90·0%)	54 (73·0%)	47 (61·8%)	598 (82·0%)	<0·0001
Any bulk oxygen source*	105 (54·1%)	105 (39·5%)	21 (30·4%)	31 (62·0%)	44 (59·5%)	35 (46·1%)	341 (46·8%)	<0·0001
Pressure-swing-adsorption plants	65 (33·5%)	64 (24·1%)	16 (23·2%)	6 (12·0%)	38 (51·4%)	25 (32·9%)	214 (29·4%)	<0·0001
Liquid oxygen	72 (37·1%)	73 (27·4%)	14 (20·3%)	30 (60·0%)	28 (37·8%)	26 (34·2%)	243 (33·3%)	<0·0001
Oxygen distribution systems								
Central or subcentral piping	75 (38·7%)	79 (29·7%)	26 (37·7%)	40 (80·0%)	48 (64·9%)	21 (27·6%)	289 (39·6%)	<0·0001
Cylinder transport	123 (63·4%)	209 (78·6%)	39 (56·5%)	44 (88·0%)	52 (70·3%)	39 (51·3%)	506 (69·4%)	<0·0001
Respiratory tubing	105 (54·1%)	169 (63·5%)	33 (47·8%)	49 (98·0%)	50 (67·6%)	46 (60·5%)	452 (62·0%)	<0·0001
Oxygen delivery devices								
Nasal cannula	143 (73·7%)	216 (81·2%)	41 (59·4%)	45 (90·0%)	59 (79·7%)	66 (86·8%)	570 (78·2%)	0·0002
Nasal catheter	123 (63·4%)	195 (73·3%)	44 (63·8%)	19 (38·0%)	46 (62·2%)	68 (89·5%)	495 (67·9%)	<0·0001
Face mask	163 (84·0%)	244 (91·7%)	63 (91·3%)	50 (100·0%)	64 (86·5%)	70 (92·1%)	654 (89·7%)	0·0051
Continuous positive airway pressure^†^	58 (29·9%)	112 (42·1%)	19 (27·5%)	7 (14·0%)	17 (23·0%)	26 (34·2%)	239 (32·8%)	0·0002
Bilevel positive airway pressure^†^	67 (34·5%)	52 (19·5%)	15 (21·7%)	16 (32·0%)	28 (37·8%)	24 (31·6%)	202 (27·7%)	0·0013
Invasive mechanical ventilation	96 (49·5%)	151 (56·8%)	24 (34·8%)	25 (50·0%)	27 (36·5%)	34 (44·7%)	357 (49·0%)	0·030
Monitoring devices								
Pulse oximeter	154 (79·4%)	232 (87·2%)	52 (75·4%)	46 (92·0%)	60 (81·1%)	62 (81·6%)	606 (83·1%)	0·042
Multiparameter monitor	116 (59·8%)	178 (66·9%)	44 (63·8%)	45 (90·0%)	38 (51·4%)	29 (38·2%)	450 (61·7%)	<0·0001
Quality assurance systems								
Concentration control	144 (74·2%)	209 (78·6%)	57 (82·6%)	49 (98·0%)	58 (78·4%)	61 (80·3%)	578 (79·3%)	0·0031
Maintenance schedule	143 (73·7%)	202 (75·9%)	50 (72·5%)	48 (96·0%)	50 (67·6%)	60 (78·9%)	553 (75·9%)	0·0027

Data are n (%). *Bulk oxygen source refers to either pressure-swing-adsorption plants or liquid oxygen. †For continuous positive airway pressure and bilevel positive airway pressure, only availability (and not equipment functionality) was assessed.

**Table 4:** Availability of functional oxygen system components in 506 tertiary-care level facilities that offer oxygen therapy, by WHO subregion

	Africa D (n=127)	Africa E (n=135)	Eastern Mediterranean D (n=46)	South-East Asian B (n=55)	South-East Asian D (n=67)	Western Pacific B (n=76)	Total (n=506)	p value
Oxygen sources								
Cylinders	111 (87·4%)	127 (94·1%)	39 (84·8%)	53 (96·4%)	67 (100·0%)	61 (80·3%)	458 (90·5%)	<0·0001
Concentrators	113 (89·0%)	127 (94·1%)	40 (87·0%)	53 (96·4%)	64 (95·5%)	51 (67·1%)	448 (88·5%)	<0·0001
Any bulk oxygen source*	100 (78·7%)	82 (60·7%)	26 (56·5%)	53 (96·4%)	59 (88·1%)	39 (51·3%)	359 (70·9%)	<0·0001
Pressure-swing-adsorption plants	77 (60·6%)	66 (48·9%)	20 (43·5%)	36 (65·5%)	55 (82·1%)	28 (36·8%)	282 (55·7%)	<0·0001
Liquid oxygen	74 (58·3%)	51 (37·8%)	16 (34·8%)	53 (96·4%)	46 (68·7%)	29 (38·2%)	269 (53·2%)	<0·0001
Oxygen distribution systems								
Central or subcentral piping	88 (69·3%)	72 (53·3%)	16 (34·8%)	52 (94·5%)	59 (88·1%)	38 (50·0%)	325 (64·2%)	<0·0001
Cylinder transport	112 (88·2%)	114 (84·4%)	25 (54·3%)	52 (94·5%)	56 (83·6%)	59 (77·6%)	418 (82·6%)	<0·0001
Respiratory tubing	100 (78·7%)	102 (75·6%)	25 (54·3%)	55 (100·0%)	59 (88·1%)	57 (75·0%)	398 (78·7%)	<0·0001
Oxygen delivery devices								
Nasal cannula	107 (84·3%)	127 (94·1%)	30 (65·2%)	53 (96·4%)	58 (86·6%)	66 (86·8%)	441 (87·2%)	<0·0001
Nasal catheter	95 (74·8%)	123 (91·1%)	29 (63·0%)	48 (87·3%)	58 (86·6%)	64 (84·2%)	417 (82·4%)	0·0002
Face mask	115 (90·6%)	129 (95·6%)	38 (82·6%)	55 (100·0%)	61 (91·0%)	67 (88·2%)	465 (91·9%)	0·0062
Continuous positive airway pressure^†^	69 (54·3%)	87 (64·4%)	16 (34·8%)	27 (49·1%)	48 (71·6%)	44 (57·9%)	291 (57·5%)	0·0012
Bilevel positive airway pressure^†^	61 (48·0%)	65 (48·1%)	23 (50·0%)	45 (81·8%)	58 (86·6%)	33 (43·4%)	285 (56·3%)	<0·0001
Invasive mechanical ventilation	89 (70·1%)	107 (79·3%)	18 (39·1%)	48 (87·3%)	50 (74·6%)	47 (61·8%)	359 (70·9%)	0·0001
Monitoring devices								
Pulse oximeter	112 (88·2%)	128 (94·8%)	43 (93·5%)	53 (96·4%)	58 (86·6%)	63 (82·9%)	457 (90·3%)	0·029
Multiparameter monitor	103 (81·1%)	118 (87·4%)	24 (52·2%)	50 (90·9%)	58 (86·6%)	47 (61·8%)	400 (79·1%)	<0·0001
Quality assurance systems								
Concentration control	104 (81·9%)	117 (86·7%)	39 (84·8%)	55 (100·0%)	61 (91·0%)	60 (78·9%)	436 (86·2%)	0·0014
Maintenance schedule	111 (87·4%)	112 (83·0%)	36 (78·3%)	55 (100·0%)	59 (88·1%)	64 (84·2%)	437 (86·4%)	0·0038

Data are n (%). *Bulk oxygen source refers to either pressure-swing-adsorption plants or liquid oxygen. †For continuous positive airway pressure and bilevel positive airway pressure, only availability (and not equipment functionality) was assessed.

## Data Availability

The authors are willing to make the source data available upon targeted inquiry to the corresponding author according to the policy and requirements of their respective institutions.

## Research in context

### Evidence before this study

We searched Ovid MEDLINE, Embase, Global Health, Health, and Psychosocial Instruments, and ERIC databases from database inception to July 12, 2023, without language restrictions, to identify literature related to facility-level oxygen system assessment. We used the following search terms: (“medical oxygen” OR “oxygen” OR “pressure-swing-adsorption” OR “PSA” OR “oxygen plant” OR “oxygen cylinder” OR “oxygen concentrator” OR “liquid oxygen”) AND (“facility readiness” OR “facility evaluation” OR “facility assessment”). We restricted our search to studies and assessments conducted in low-income and middle-income countries as defined by the World Bank. We retrieved 32 full-text articles from our search that were relevant to facility-level assessments of oxygen availability or use. We supplemented these articles with eight country-level facility oxygen system assessments known to The Global Fund to Fight AIDS, Tuberculosis and Malaria from pre-existing multilateral partnerships. This previous literature revealed that facility-level assessments of oxygen systems in low-income and middle-income countries have largely focused on oxygen access in single countries or facility types (eg, facilities providing COVID-19 care). There was a lack of standardised data across countries, facility levels, and facility types characterising reliable oxygen availability and the functional availability of specific oxygen system components.

### Added value of this study

To the best of our knowledge, this study is the first large, multinational, standardised, facility-level survey assessing oxygen systems in low-income and middle-income countries. We assessed not only oxygen availability, but also the reliable oxygen availability and the functional availability of 17 specific oxygen system components across three facility care levels and six WHO subregions. We found a staggering scarcity of reliable oxygen availability across care levels and geographical subregions as well as substantial inequities in reliable oxygen availability and functional availability of almost all measured system components across subregions.

### Implications of all the available evidence

Combined with previous literature, our findings highlight the need for a standardised approach to oxygen system measurement at different facility care levels, sub-facility-level research on the implementation of specific oxygen system components, and analyses of the structural, financial, technical, and political drivers of systemic inequities.
