# Supplementary material for: A rapid facility-level assessment of oxygen systems in 39 low-income and middle-income countries: a cross-sectional study
Source: Lancet Glob Health. Author manuscript; Available in PMC 2025 Apr 1. (PMC11954661; doi:10.1016/S2214-109X(24)00561-8)
Supplement: Supplementary appendix 1 [file NIHMS2062996-supplement-Supplementary_appendix_1.pdf]

THE LANCET
Global Health

## Supplementary appendix 1

This translation in French was submitted by the authors and we reproduce it as supplied. It has not been peer reviewed. *The Lancet*'s editorial processes have only been applied to the original in English, which should serve as reference for this manuscript.

Cette traduction en français a été proposée par les auteurs et nous l'avons reproduite telle quelle. Elle n'a pas été examinée par des pairs. Les processus éditoriaux du *Lancet* n'ont été appliqués qu'à l'original en anglais et c'est cette version qui doit servir de référence pour ce manuscrit.

Supplement to: Ijaz N, Lee T, Furtado N, et al. A rapid facility-level assessment of oxygen systems in 39 low-income and middle-income countries: a cross-sectional study. *Lancet Glob Health* 2025; published online Feb 27. https://doi.org/10.1016/S2214-109X(24)00561-8.

**Contexte :** L'inégalité d'accès à l'oxygène médical est l'un des principaux facteurs d'inégalités mondiales de morbidité et de mortalité. Notre objectif était de décrire la fiabilité de la disponibilité de l'oxygène (i.e., quand la disponibilité est ininterrompue) et la disponibilité fonctionnelle du système d'oxygène (i.e., quand les éléments du système d'oxygène sont en état de marche) dans 39 pays à revenu faible et intermédiaire, et à comparer entre les sous-régions de l'OMS.

**Méthodes :** Nous présentons les données d'une enquête transversale provenant d'établissements de santé de niveau primaire, secondaire et tertiaire, dans six sous-régions de l'OMS. Les établissements ont été sélectionnés par un échantillonnage ciblé et aléatoire stratifié. Les enquêteurs ont visité les établissements entre septembre 2022 et février 2023 pour soumettre un questionnaire standardisé aux directeurs des établissements. Tous les établissements contactés ont répondu. Les questions ont évalué la fiabilité de la disponibilité de l'oxygène au cours des 3 mois précédents et la disponibilité fonctionnelle des éléments du système : sources d'oxygène (i.e., cylindres, concentrateurs, générateurs d'oxygène et oxygène liquide), systèmes de distribution (tuyauterie, transport de cylindres et tubulures respiratoires), dispositifs d'administration de l'oxygène aux patients (i.e., interfaces nasales, masques, et autres dispositifs), dispositifs de surveillance (i.e., oxymètres de pouls et moniteurs multiparamétriques) et assurance qualité (i.e., contrôle de la concentration en oxygène et programme de maintenance). Nous rapportons des statistiques descriptives et comparons les sous-régions à l'aide du $\chi^2$ et test exact de Fisher.

**Résultats :** Sur 2 884 établissements interrogés, 304 (24,5 %) des 1 241 établissements primaires, 558 (52,4 %) des 1 064 établissements secondaires et 387 (66,8 %) des 579 établissements tertiaires ont rapporté que la disponibilité de l'oxygène était fiable. Les établissements de tous les niveaux et de toutes les sous-régions manquaient de certains éléments du système, avec des différences statistiquement significatives ($p < 0.05$) pour la disponibilité fonctionnelle de chacun des éléments du système dans les sous-régions, à tous les niveaux. Par exemple, la disponibilité fonctionnelle des cylindres variait de 56,7 % à 100,0 %, de la tuyauterie de 7,5 % à 94,6 %, des canules nasales de 56,3 % à 96,4 %, et des oxymètres de pouls de 47,8 % à 96,4 %, selon le niveau et la sous-région.

**Interprétation :** La fiabilité de la disponibilité de l'oxygène était faible dans tous les niveaux d'établissement et pour toutes les sous-régions. Il y avait des disparités significatives pour la disponibilité fonctionnelle des éléments du système d'oxygène dans les sous-régions, avec d'importantes implications pour l'équité et le financement de la santé mondiale.
