# Supplementary material for: A rapid facility-level assessment of oxygen systems in 39 low-income and middle-income countries: a cross-sectional study
Source: Lancet Glob Health. Author manuscript; Available in PMC 2025 Apr 1. (PMC11954661; doi:10.1016/S2214-109X(24)00561-8)
Supplement: Supplementary Appendix 3 [file NIHMS2062996-supplement-Supplementary_Appendix_3.pdf]

THE LANCET
Global Health

Supplementary appendix 3

This Equitable Partnership Declaration (EPD) was submitted by the authors, and we reproduce it as supplied. It has not been peer reviewed. *The Lancet*'s editorial processes have not been applied to the EPD.

Supplement to: Ijaz N, Lee T, Furtado N, et al. A rapid facility-level assessment of oxygen systems in 39 low-income and middle-income countries: a cross-sectional study. *Lancet Glob Health* 2025; published online Feb 27. https://doi.org/10.1016/S2214-109X(24)00561-8.

Equitable Partnership Declaration questions

This Equitable Partnership Declaration is a statement being published online alongside papers at *The Lancet Global Health*, as a separate appendix, to allow researchers to describe how their work engages with researchers, communities, and environments in the countries of study. This is part of our broader goal to decolonise global health, handing control and leadership of research to academics and clinicians who are based in the regions of study, and to affected communities.

Please answer all questions with as much detail as possible, noting that all included information will be published open-access and it will be freely available online to all who wish to read it. If a question does not apply to your study, please state "Not applicable".

The format of and questions in this statement are currently in a pilot phase. Please email Dr Kate McIntosh (Kate.McIntosh@lancet.com; deputy editor) with any feedback, particularly if you find any questions unclear.

Researcher considerations

1. Please detail the involvement that researchers who are based in the region(s) of study had during a) study design; b) clinical study processes, such as processing blood samples, prescribing medication, or patient recruitment; c) data interpretation; and d) manuscript preparation, commenting on all aspects. If they were not involved in any of these aspects, please explain why.

   This question is intended for international partnerships; if all your authors are based in the area of study, this question is not applicable.

   This should include a thorough description of their leadership role(s) in the study. Are local researchers named in the author list or the acknowledgements, or are they not mentioned at all (and, if not, why)? Please also describe the involvement of early career researchers based in the location of the study. Some of this information might be repeated from the Contributors section in the manuscript. Note: we adhere to ICMJE authorship criteria when deciding who should be named on a paper.

**a) Study design:** Because this survey was conducted in many countries for Global Fund internal quality assurance and program monitoring purposes, the study design was developed by the Global Fund team to ensure standardized data collection across countries.
**b) Clinical study processes:** There were no clinical study processes.
**c) Data interpretation:** All researchers involved in data interpretation were based at either the Global Fund or Yale University.
**d) Manuscript preparation:** All researchers involved in manuscript preparation were based at either the Global Fund or Yale University.

2. Were the data used in your study collected by authors named on the paper, or have they been extracted from a source such as a national survey? ie, is this a secondary analysis of data that were not collected by the authors of this paper. If the authors of this paper were not involved in data collection, how were data interpreted with sufficient contextual knowledge?

   The Lancet Global Health *believe contextual understanding is crucial for informed data analysis and interpretation.*

The data were collected by three data service providers (IQVIA, KPMG, and McKinsey) contracted by the Global Fund to collect the data following a standardized protocol.<br><br>While the authors were not involved in data collection, many of us have worked in relevant clinical and public health contexts, informing our data analysis and interpretation:<br><br>Dr. Nadir Ijaz has worked clinically at a government hospital in Pakistan and conducted research, including primary data collection, at health facilities in Pakistan, India, and Bangladesh. He also completed four months of his clinical residency training in Pakistan.<br><br>Dr. Emilie Macher has served as a medical doctor or medical team lead in field projects based in multiple countries included in this study. Later, as headquarter staff, she worked with Médecins Sans Frontières and supported numerous medical projects or emergency responses in several Sub-Saharan African countries between 2009 and 2020. Since 2022, with the Global Fund, she provides technical support to the implementation of COVID-19 Response Mechanism oxygen-related investments in 14 countries including Benin, Burkina Faso, Cameroon, Chad, DRC, Guinea, Madagascar, Mali, Niger, Senegal and Togo.<br><br>Zipporah Muitheri has worked in Kenya for 10 years in various capacities, including as nursing officer in several hospitals, training healthcare providers and pre-service students on medical oxygen administration, and as Quality Assurance Officer for donor funded programs (HIV/AIDS, TB, Malaria, RMNCH).<br><br>Dr. Benjamin Park has been working on international and global public health for 15+ years, in over 30 of the countries included in this study.<br><br>Dr. David Lowrance has worked and lived in Myanmar, Rwanda, Namibia, Haiti and Tanzania over the past 20 years. In addition, he has worked in Cameroon, Ghana, Malawi, Mozambique, Sierra Leone, and Zambia.

3. How was funding used to remunerate and enhance the skills of researchers and institutions based in the area(s) of study? And how was funding used to improve research infrastructure in the area of study?

   Potentially effective investments into long-term skills and opportunities within institutions could include training or mentorship in analytical techniques and manuscript writing, opportunities to lead all or specific aspects of the study, financial remuneration rather than requiring volunteers, and other professional development and educational opportunities.

Improvements to research infrastructure could be funding of extended trial designs (such as platform trials) and use of master protocols to enable these designs, establishment of long-term contracts for research staff, building research facilities, and local control of funding allocation.

**Skills:** This study did not provide direct funding to enhance the skills of local researchers and institutions. However, funding provided to data service providers may have indirectly enhanced skills of local public health staff through providing additional training on conducting facility assessments.
**Research infrastructure:** This study did not provide funding to improve local research infrastructure.

4. How did you safeguard the researchers who implemented the study?

Please describe how you guaranteed safe working conditions for study staff, including provision of appropriate personal protective equipment, protection from violence, and prevention of overworking.

The Global Fund excluded any health facilities located in unsafe geographic areas (e.g., conflict zones) from sampling in this study to ensure safe working conditions for study staff.

Benefits to the communities and regions of study

5. How does the study address the research and policy priorities of its location?

How were the local priorities determined and then used to inform the research question? Who decided which priorities to take forward? Which elements of the study address those priorities?

The Global Fund partnered closely with country ministries of health to conduct this survey and determine local priorities. The inclusion of both facilities financed and not financed by the Global Fund was an important element of the study design that centred country priorities.

6. How will research products be shared in the community of study?

For instance, will you be providing written or oral layperson summaries for non-academic information sharing? Will study data be made available to institutions in the region(s) of study? The Lancet Global Health *encourages authors to translate the summary (abstract) into relevant languages after paper editing; do you intend to translate your summary?*

The Global Fund has a well-developed process for data dissemination to country ministries of health and will share country-level data from the larger survey as per policy and mutual agreements with each ministry of health. Study data may additionally be made available to institutions in the countries of study upon request, according to the policies and requirements of the Global Fund.

Many of the countries included in our study are primarily French-speaking, and we therefore intend to provide a French translation of our summary after paper editing.

7. How were individuals, communities, and environments protected from harm?

a) *How did you ensure that sensitive patient data was handled safely and respectfully? Was there any potential for stigma or discrimination against participants arising from any of the procedures or outcomes of the study?*

We did not collect sensitive patient data.

b) *Might any of the tests be experienced as invasive or culturally insensitive?*

We did not conduct any tests.

c) *How did you determine that work was sensitive to traditions, restrictions, and considerations of all cultural and religious groups in the study population?*

We do not expect that local traditions, restrictions, and other cultural or religious considerations were relevant in our assessment of health facilities.

d) *Were biowaste and radioactive waste disposed of in accordance with local laws?*

Not applicable.

e) *Were any structures built that would have impacted members of the community or the environment (such as handwashing facilities in a public space)? If so, how did you ensure that you had appropriate community buy-in?*

Not applicable.

f) *How might the study have impacted existing health-care resources (such as staff workloads, use of equipment that is typically employed elsewhere, or reallocation of public funds)?*

As this was an observational study that only required a meeting with local administrative facility leadership, we do not anticipate that it significantly impacted staff workload or other healthcare resources.

8. Finally, please provide the title (eg, Dr/Prof, Mr/Mrs/Ms/Mx), name, and email address of an author who can be contacted about this statement. This can be the corresponding author.

Name: Dr. Nadir Ijaz
Email: nadir.ijaz@yale.edu
