# Supplementary material for: A rapid facility-level assessment of oxygen systems in 39 low-income and middle-income countries: a cross-sectional study
Source: Lancet Glob Health. Author manuscript; Available in PMC 2025 Apr 1. (PMC11954661; doi:10.1016/S2214-109X(24)00561-8)
Supplement: Supplementary Appendix 2 [file NIHMS2062996-supplement-Supplementary_Appendix_2.pdf]

THE LANCET
Global Health

Supplementary appendix 2

This appendix formed part of the original submission and has been peer reviewed. We post it as supplied by the authors.

Supplement to: Ijaz N, Lee T, Furtado N, et al. A rapid facility-level assessment of oxygen systems in 39 low-income and middle-income countries: a cross-sectional study. *Lancet Glob Health* 2025; published online Feb 27. https://doi.org/10.1016/S2214-109X(24)00561-8.

Supplementary materials: Reliable oxygen availability and functional availability of oxygen system components by facility care level and geographic subregion in 39 low- and middle-income countries: A cross-sectional study

Appendix 1. STROBE checklist. 2
Appendix 2. Data collection by subregion and study month...6
Appendix 3. Questionnaire. 7

Appendix 1. STROBE checklist.

STROBE Statement—checklist of items that should be included in reports of observational studies.

	Item No.	Recommendation	Page No.	Relevant text from manuscript
**Title and abstract**	1	(*a*) Indicate the study's design with a commonly used term in the title or the abstract	3	"A cross-sectional study"
		(*b*) Provide in the abstract an informative and balanced summary of what was done and what was found	4-5	
**Introduction**				
Background/rationale	2	Explain the scientific background and rationale for the investigation being reported	5-6	
Objectives	3	State specific objectives, including any prespecified hypotheses	6-7	"Specifically, we aim to describe facility-level *reliable oxygen availability* and the *functional availability* of specific types of oxygen sources, distribution systems, delivery devices, and hypoxemia monitoring devices, stratified by facility care level and World Health Organization (WHO) subregion, and to compare these across WHO subregions."
**Methods**				
Study design	4	Present key elements of study design early in the paper	7	
Setting	5	Describe the setting, locations, and relevant dates, including periods of recruitment, exposure, follow-up, and data collection	7	
Participants	6	(*a*) *Cohort study*—Give the eligibility criteria, and the sources and methods of selection of participants. Describe methods of follow-up<br>*Case-control study*—Give the eligibility criteria, and the sources and methods of case ascertainment and control selection. Give the rationale for the choice of cases and controls<br>*Cross-sectional study*—Give the eligibility criteria, and the sources and methods of selection of participants	7-9	
		(*b*) *Cohort study*—For matched studies, give matching criteria and number of exposed and unexposed<br>*Case-control study*—For matched studies, give matching criteria and the number of controls per case	n/a	
Variables	7	Clearly define all outcomes, exposures, predictors, potential confounders, and effect modifiers. Give diagnostic criteria, if applicable	9-11	
Data sources/ measurement	8*	For each variable of interest, give sources of data and details of methods of assessment (measurement). Describe comparability of assessment methods if there is more than one group	9-11	

Bias	9	Describe any efforts to address potential sources of bias	11
Study size	10	Explain how the study size was arrived at	7-9

Continued on next page

Quantitative variables	11	Explain how quantitative variables were handled in the analyses. If applicable, describe which groupings were chosen and why	10-11
Statistical methods	12	(*a*) Describe all statistical methods, including those used to control for confounding	10-11
		(*b*) Describe any methods used to examine subgroups and interactions	n/a
		(*c*) Explain how missing data were addressed	n/a
		(*d*) *Cohort study*—If applicable, explain how loss to follow-up was addressed<br>*Case-control study*—If applicable, explain how matching of cases and controls was addressed<br>*Cross-sectional study*—If applicable, describe analytical methods taking account of sampling strategy	11
		(*e*) Describe any sensitivity analyses	n/a
**Results**			
Participants	13*	(a) Report numbers of individuals at each stage of study—eg numbers potentially eligible, examined for eligibility, confirmed eligible, included in the study, completing follow-up, and analysed	11-12
		(b) Give reasons for non-participation at each stage	n/a
		(c) Consider use of a flow diagram	n/a
Descriptive data	14*	(a) Give characteristics of study participants (eg demographic, clinical, social) and information on exposures and potential confounders	11-14
		(b) Indicate number of participants with missing data for each variable of interest	n/a
		(c) *Cohort study*—Summarise follow-up time (eg, average and total amount)	
Outcome data	15*	*Cohort study*—Report numbers of outcome events or summary measures over time	
		Case-control study—Report numbers in each exposure category, or summary measures of exposure	
		Cross-sectional study—Report numbers of outcome events or summary measures	11-14
Main results	16	(*a*) Give unadjusted estimates and, if applicable, confounder-adjusted estimates and their precision (eg, 95% confidence interval). Make clear which confounders were adjusted for and why they were included	11-14
		(*b*) Report category boundaries when continuous variables were categorized	n/a
		(*c*) If relevant, consider translating estimates of relative risk into absolute risk for a meaningful time period	n/a

Continued on next page

Other analyses	17	Report other analyses done—eg analyses of subgroups and interactions, and sensitivity analyses	n/a
**Discussion**			
Key results	18	Summarise key results with reference to study objectives	14
Limitations	19	Discuss limitations of the study, taking into account sources of potential bias or imprecision. Discuss both direction and magnitude of any potential bias	16-18
Interpretation	20	Give a cautious overall interpretation of results considering objectives, limitations, multiplicity of analyses, results from similar studies, and other relevant evidence	14-16
Generalisability	21	Discuss the generalisability (external validity) of the study results	16-18
**Other information**			
Funding	22	Give the source of funding and the role of the funders for the present study and, if applicable, for the original study on which the present article is based	11

*Give information separately for cases and controls in case-control studies and, if applicable, for exposed and unexposed groups in cohort and cross-sectional studies.

Note: An Explanation and Elaboration article discusses each checklist item and gives methodological background and published examples of transparent reporting. The STROBE checklist is best used in conjunction with this article (freely available on the Web sites of PLoS Medicine at http://www.plosmedicine.org/, Annals of Internal Medicine at http://www.annals.org/, and Epidemiology at http://www.epidem.com/). Information on the STROBE Initiative is available at www.strobe-statement.org.

Appendix 2. Data collection by subregion and study month.

The following table shows when facility assessments were completed in each subregion by study month.

Subregion	Sept 2022	Oct 2022	Nov 2022	Dec 2022	Jan 2023	Feb 2023	Total
**AFR D**	255	530	155	0	0	0	940
**AFR E**	216	634	41	0	0	0	891
**EMR D**	87	108	0	14	53	11	273
**SEAR B**	48	186	0	0	0	0	234
**SEAR D**	69	140	25	0	0	0	234
**WPR B**	100	201	11	0	0	0	312
**Total**	775	1799	232	14	53	11	2884

Appendix 3. Questionnaire.

The following table shows the Oxygen Readiness Section of the Supply Chain and Health Services Spot Check questionnaire.

Question Code	Description of Variable	Question	Admissable Values	Additional Details	Type of Question
2.3.1.1	Oxygen therapy and related service	Does the facility offer oxygen therapy and related services?	Yes<br>No	*If "No," skip to question number 4.2.3.1.4 (non-invasive ventilation)*	Single choice
2.3.1.2	Oxygen therapy available	Is oxygen therapy available on the day of the visit for the following components of source, distribution, delivery and monitoring?			
2.3.1.2.1	Source: Cylinders	Cylinders	Yes<br>No		Single choice
2.3.1.2.2	Source: Concentrator	Concentrators	Yes<br>No		Single choice
2.3.1.2.3	Source: Oxygen PSA plant	Oxygen PSA (pressure swing adsorption) plant	Yes<br>No		Single choice
2.3.1.2.4	Source: Liquid Oxygen	Liquid Oxygen	Yes<br>No		Single choice
2.3.1.2.5	Distribution: Central or sub central piping	Central or sub central piping	Yes<br>No		Single choice
2.3.1.2.6	Distribution: Transport for cylinders	Transport for cylinders	Yes<br>No		Single choice
2.3.1.2.7	Distribution: Tubing	Tubing	Yes<br>No		Single choice
2.3.1.2.8	Delivery: Nasal cannula (prongs)	Nasal cannula (prongs)	Yes<br>No		Single choice
2.3.1.2.9	Delivery: Nasal catheter	Nasal catheter	Yes<br>No		Single choice
2.3.1.2.10	Delivery: Face mask	Face mask	Yes		Single choice

			No	
2.3.1.2.11	Monitoring: Pulse oximeter	Pulse oximeter	Yes<br>No	Single choice
2.3.1.2.12	Monitoring: Multiparameter monitor	Multiparameter monitor	Yes<br>No	Single choice
2.3.1.3	Oxygen therapy functional	If oxygen therapy is available, are the following components of source, distribution, delivery and monitoring functional?		
2.3.1.3.1	Source: Cylinders	Cylinders	Yes<br>No	Single choice
2.3.1.3.2	Source: Concentrator	Concentrators	Yes<br>No	Single choice
2.3.1.3.3	Source: Oxygen PSA plant	Oxygen PSA plant	Yes<br>No	Single choice
2.3.1.3.4	Source: Liquid Oxygen	Liquid Oxygen	Yes<br>No	Single choice
2.3.1.3.5	Distribution: Central or sub central piping	Central or sub central piping	Yes<br>No	Single choice
2.3.1.3.6	Distribution: Transport for cylinders	Transport for cylinders	Yes<br>No	Single choice
2.3.1.3.7	Distribution: Tubing	Tubing	Yes<br>No	Single choice
2.3.1.3.8	Delivery: Nasal cannula (prongs)	Nasal cannula (prongs)	Yes<br>No	Single choice
2.3.1.3.9	Delivery: Nasal catheter	Nasal catheter	Yes<br>No	Single choice
2.3.1.3.10	Delivery: Face mask	Face mask	Yes<br>No	Single choice
2.3.1.3.11	Monitoring: Pulse oximeter	Pulse oximeter	Yes<br>No	Single choice
2.3.1.3.12	Monitoring: Multiparameter monitor	Multiparameter monitor	Yes<br>No	Single choice

2.3.1.4	Non-invasive ventilation equipment available	Does the facility have non-invasive ventilation equipment, e.g. bi-PAP, CPAP?	Yes, bi PAP<br>Yes, CPAP<br>Yes, both<br>No<br>Do not know		Single choice
2.3.1.5	Mechanical ventilation equipment available	Does the facility have mechanical ventilation equipment?	Yes<br>No<br>Do not know	*If "No" or "Do not know," skip next question*	Single choice
2.3.1.6	Mechanical ventilation equipment total available and functional	How many units in total and functional of mechanical ventilation equipment?			
2.3.1.6.1	Total units of ventilation equipment	Total	________(###)		Free text
2.3.1.6.2	Functional units of ventilation equipment	Functional	________(###)		Free text
2.3.1.7	Oxygen unavailable at any time during the past 3 months	Has oxygen been unavailable in this facility at any time during the past 3 months for any reason?	Yes<br>No<br>Do not know		Single choice
2.3.2.1	Maintenance of O2 equipment	Does your facility implement a maintenance schedule for critical equipment generating O2?	Yes<br>No		Single choice
2.3.2.2	O2 concentration controls	Is control of O2 concentration for main equipment regularly implemented?	Yes<br>No		Single choice
2.3.2.3	Inspection and fit testing	Is inspection and fit testing regularly performed before using disposable, reusable masks and respiratory protective equipment?	Yes<br>No		Single choice
